# Molecular docking and antihypertensive effects of a novel angiotensin-I converting enzyme inhibitory peptide from yak bone

**DOI:** 10.3389/fnut.2022.993744

**Published:** 2022-10-12

**Authors:** Xinchang Gao, Fan Bu, Dalong Yi, Huaigao Liu, Zhiying Hou, Chaoying Zhang, Chang Wang, Jin-Ming Lin, Yali Dang, Yufen Zhao

**Affiliations:** ^1^Department of Chemistry, Tsinghua University, Beijing, China; ^2^Institute of Drug Discovery Technology, Ningbo University, Ningbo, China; ^3^College of Food and Pharmaceutical Sciences, Ningbo University, Ningbo, China; ^4^Anhui Guotai Biotechnology Co., Ltd., Xuancheng, China

**Keywords:** molecular docking, antihypertensive effects, yak bone, ACE inhibitory peptide, digestion

## Abstract

A novel angiotensin-converting enzyme (ACE) inhibitory peptide ser-ala-ser-val-ile-pro-val-ser-ala-val-arg-ala (SASVIPVSAVRA) was purified and identified from yak bone by Electrospray Ionization-Time of Flight-Mass Spectrometry (ESI-TOF-MS). Results *in vitro* showed that the peptide exhibited strong ACE inhibition activities with an IC_50_ of 54.22 μM. Molecular docking results showed the binding between the peptide SASVIPVSAVRA and ACE mainly driven by van der Waals forces, hydrogen bonds and metal receptor. Interestingly, the ACE inhibition activities of the peptide increased about 19% after digestion, but none of its metabolites showed stronger activity than it. The *in vivo* experiment showed that the antihypertensive effect of peptide SASVIPVSAVRA at dose of 30 mg/kg is nearly equal to Captopril at dose of 10 mg/kg to spontaneously hypertensive rats (SHRs). The antihypertensive effect mechanism of SASVIPVSAVRA should be further studied through plasma metabolomics and bioanalysis. Structure analysis of amino acids and peptides produced during digestion may help better understand the antihypertensive effect of peptides.

## Graphical Abstract

Study on the peptide ser-ala-ser-val-ile-pro-val-ser-ala-val-arg-ala (SASVIPVSAVRA) *in vitro* and *in vivo*. 
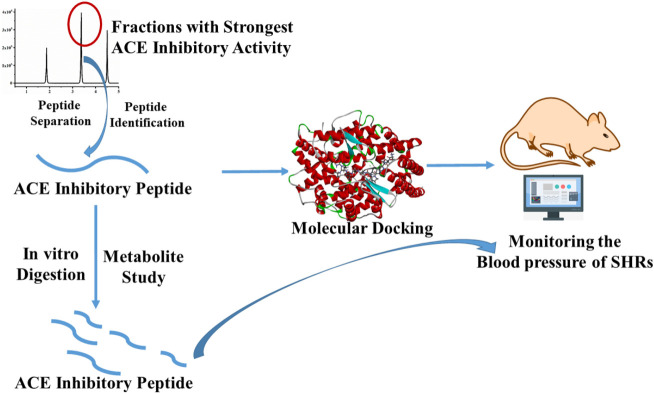

## Introduction

Hypertension is the most common chronic disease and a significant threat to cerebrovascular diseases. It usually causes headaches, inattention, memory loss, chest tightness, fatigue, and other symptoms (1).

With the continuous increase in the number of people suffering from hypertension, researchers have paid more attention to the prevention, and the treatment of hypertension. The renin-angiotensin system (RAS) and kallikrein-kinin system (KKS) play an important role in regulating blood pressure *in vivo* (2). In RAS, Renin cleaves angiotensinogen to produce the inactive Angiotensin I, and through the action of ACE, which generated Angiotensin II with vascular smooth muscle activity, thereby causing the blood pressure spiked. In KKS, ACE deactivates bradykinin, and bradykinin stabilizes blood pressure by relaxation blood vessels and regulating electrolytes. Unfortunately, synthetic ACE inhibitors (captopril and enalapril) occasionally cause some adverse reactions, such as coughing, allergic reactions, and elevated blood potassium levels (3). More and more attention has been focused on seeking food-derived ACE inhibitory peptides that could lower blood pressure and reduce side effects in recent years. Interestingly, more and more reports appeared about the natural polypeptides with potential ACE inhibitory activity *in vitro* and *in vivo*, and they are structurally similar to synthetic drugs (4). These ACE inhibitors are safe without side effects.

Animal bones are an important source of functional peptide (5, 6). Researchers found many food-derived antihypertensive peptides in animals and plants, including fungi, milk, yeast, cheese, fish, and soybeans (7, 8). The ACE inhibitory activities of peptides are nearly related to their unique amino acid composition, such as Pro, Ala, and Val (9). Interestingly, yak bones are also rich in these unique amino acids. Chen et al. (10) reported that yak bones were rich in glycine, alanine, proline and glutamic acid (10). However, there are few reports on the isolation of ACE inhibitory peptide sequences from yak bones. Sun et al. (11) reported an antioxidant peptide GPHGAAGVA from yak bone. Ye et al. (12) reported an peptide GPSGPAGKDGRIGQPG from Yak bone with osteoblast-proliferating activity.

This study aims to purify and identified the bioactive peptides from Yak bone, evaluate the antihypertensive effects *in vivo*, explain the mechanism between peptide and ACE.

## Materials and methods

### Materials

Yak bone was provided by Anhui Guotai Biotechnology Co., Ltd., Papain (800 U/g proteins), pepsin (25,000 U/mL), pancreatin (800 U/mL), ACE, Hippuryl-histidyl-leucine (HHL), BCA Protein Assay Kit and Sephadex G-25 were purchased from Sigma-Aldrich (St. Louis, MO, USA). All the reagents were of analytical grade.

### Preparation of yak bone peptide

One hundred and fifty gram of yak bone powder was dispersed in distilled water with the mass ratio of 1:30, and the content of yak bone protein was 197 g/kg. The hydrolyzed were mixed with papain (800 U/g proteins) at 60°C, 80 r/min for 6 h. After inactiving the enzyme by boiling for 10 min, The mixture was filtered and lyophilized (13).

### Peptide separation and purification

The method of Separation and Purification was according to the method of Bu et al. (14) with minor modification (14). The lyophilized samples of the hydrolysate product were dissolved in distilled water to reach 20 mg/mL concentration.

### Peptides identification and synthesis

ESI-TOF-MS was used for the identification of peptides from HPLC Fractions as described by Dang et al. (15). Electrospray ionization (ESI) was used in positive ion mode, source temperature was set at 150°C, desolvation temperature was set at 550°C, scan range was m/z 50–1200. The most active fraction (F6) was resuspended in water at a concentration of 1 mg/mL. The filtrate was then analyzed with the ESI-TOF mass spectrometer of the ACQUITY UPLC H-Class HPLC system (Waters, USA). The column used was BEH300 C18 (2.1 × 100 mm; 1.7 μm) (Waters, USA), the flow rate was 0.2 mL/min, and the injection volume was 10 μL. All MS and MS/MS spectra were collected and analyzed using Mass Lynx (Waters version 4.1). The PepSeq program of Biolynx (Waters Corp.) software was used for sequencing. These peptides were custom-synthesized by Sangon Biotech Co., Ltd., (Shanghai, China) (16).

The purity of these peptides was above 99%.

### Determination of angiotensin-converting enzyme inhibitory activity

The original ACE Inhibitory Activity method was developed by Cushing and Cheung (17). The ACE inhibitory activity assay used here were performed according to the method of Dang et al. (15). 80 μL of 5 mM HHL (in HEPES buffer containing 300 mM NaCl at pH 8.3) and 30 μL samples were mixed in 37°C water bath for 5 min, 40 μL ACE (0.025 U/mL) was added for incubation for 1 h, The reaction was stopped by adding 250 μL of 1 M HCl. they were filtered and measured the ACE inhibitory activity by an Agilent 1260 HPLC system (Agilent Technologies, Karlsruhe, Germany).


(1) $$\mathrm{ACE}\,\mathrm{Inhibitory}\,\mathrm{activity}\%=\frac{\mathrm{Control}-\mathrm{Sample}}{\mathrm{Control}-\mathrm{Control}\,\mathrm{Blank}}$$

### *In vitro* gastrointestinal digestion

Gastrointestinal digestion was evaluated according to the method of Dang et al. (15) and Mulet-Cabero et al. (18) with minor modification. Simulated gastric juice (pH 2.0) and simulated intestinal fluid mixture (pH 7.6) were added to peptide solution and incubated at 37°C for 2 h, respectively.

### Antihypertensive effect of angiotensin-converting enzyme inhibitory peptide *in vivo*

The systolic blood pressure (SBP) changes in the SHRs were measured by the tail-cuff method to determine the antihypertensive effect of ser-ala-ser-val-ile-pro-val-ser-ala-val-arg-ala (SASVIPVSAVRA) *in vivo*. Twenty female SHRs, weighing 230 ± 15 g and the SBP exceeding 190 mmHg, were obtained from Beijing Vital River Laboratory Animal Technology Co., Ltd. (China).

The SHRs were free to eat and drink at 25 ± 4°C and a 40 ± 6% humidity. To get the rats to adapt to the environment, the systolic blood pressure was measured once a day for 1 week. After a week of adaptation, the SHRs were randomly divided into three groups with each group containing 6 SHRs: the control group (sterilized water), captopril group (10 mg/kg captopril BW), SASVIPVSAVRA (30 mg/kg SASVIPVSAVRA BW). The effect of ACE inhibitors on SBP were measured by the Softron BP non-invasive blood pressure monitor (Softron BP-2000, Tokyo, Japan) at 0, 2, 4, and 6 h after oral administration (1), The measurements were repeated five times, the recordings were reported as mean ± standard deviation (SD). All animal procedures used in this trial were approved by the Institutional Animal Care and Use Committee of Hangzhou Medical College (Approval No. ZJCLA-IACUC-20010014).

### Angiotensin-converting enzyme inhibition pattern

The ACE inhibition pattern was similar to that used by Zhang et al. (19) with slight modifications. The concentration of HHL was changed to 0.5, 1, and 2 mmol/L, and the concentration of the purified peptide was changed to 0.1 and 0.2 mg/mL.

### Molecular docking

The molecular docking was performed using the Discovery Studio software package (Neo Trident Technology Ltd., China) according to the method of Chen et al. (20) with some modifications. The crystal structure of the human ACE-lisinopril complex (1O8A.pdb) was from RCSB PDB Protein Data Bank.^1^ SBD_Site_Sphere was X: 37.8546, Y: 38.1941, Z: 47.2396 and the docking radius was 20 Å.

### Statistical analysis

All data were analyzed using SPSS for Windows Version 25.0. The results were based on the mean ± standard deviation of at least three determinations. A *P*-value of less than 0.05 was considered statistically significant.

## Results and discussion

### Isolation and purification of angiotensin-converting enzyme peptides

**FIGURE 1:**
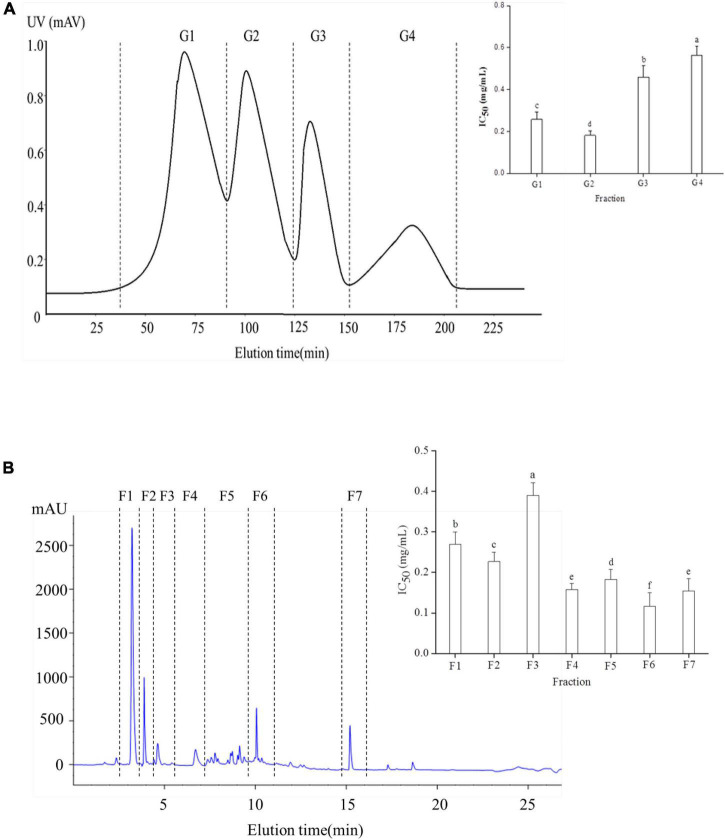
Separation and purification of a novel ACE inhibitory peptide. **(A)** Sephadex G-25 column chromatography separation diagram of Yak bone hydrolysate. **(B)** The fraction G2 purified using RP-HPLC. Different letters indicate significant differences in peptide activity (*p* < 0.05).

Results showed that the yak bone peptide prepared by section “Preparation of yak bone peptide” had ACE inhibitory activity and its IC_50_ was 1.47 mg/mL. So the ACE fractions were further separated and purified. Four components of Yak bone peptide were separated by Sephadex G-25 column (Figure 1A). G2 had the highest ACE inhibitory activity with IC_50_ value of 0.181 mg/mL, while IC_50_ values of other fractions G1, G3, and G4 were 0.258, 0.459, and 0.563 mg/mL, respectively. So, G2 was chosen for further purification, and the peptides were separated using RP-HPLC (Figure 1B). Finally, the 7 fractions were collected, and F6 had the highest ACE inhibitory activity among them(IC_50_ = 0.12 mg/mL). So F6 was collected for further experiment.

### Peptides identification

**FIGURE 2:**
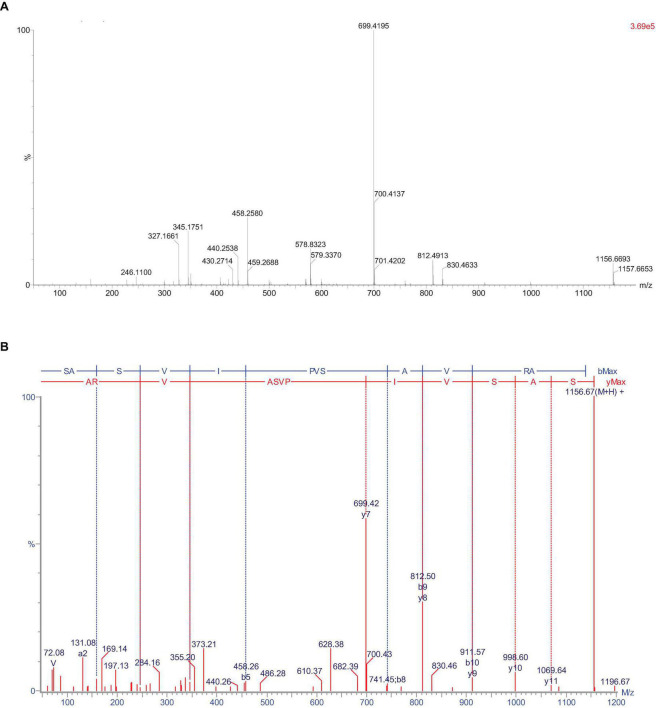
The mass spectrum of the peptide SASVIPVSAVRA. **(A)** MS spectrum **(B)** MS/MS spectrum.

Peptides from F6 was identified by ESI-TOF-MS and synthesized by the Sangon Biotech Co., Ltd. The sequence was SASVIPVSAVRA, The IC_50_ of ACE inhibitory activity was 54.22 μM, The mass spectrum of this peptide was shown in Figures 2A,B.

It was found that the IC_50_ value of SASVIPVSAVRA (54.22 μM) was much higher than the reported ACE peptide SGGSYADELVSTAK (0.093 μM) (21) while lower than other peptides, such as QLLLQQ (IC_50_, 75.0 μM) and IVVE (IC_50_, 315.3 μM) (22, 23). Therefore, it was meaningful to study the ACE inhibitory activity of SASVIPVSAVRA.

The ACE inhibitory Activity of peptides was closely related to hydrophobic amino acid residues, such as Proline, Valine, Serine, Alanine, Leucine and Isoleucine (24). Therefore, the rich hydrophobic amino acids in the peptide SASVIPVSAVRA may be the main reason for the ACE inhibitory activity. The presence of proline in the sequence may allowed the carboxyl group to interact with the positively charged residues in the active site of the enzymec to contribute to its ACE activity (25).

Generally, the presence of amino acids (Val, Ile) with hydrophobic branches would increase the ACE inhibitory activity (26). Therefore, the Val residue and Ile residue in SASVIPVSAVRA may contribute to the ACE activity of the peptide. Besides, Matsumura et al. (27) reported that the tripeptide VIP extracted from Bonito Bowels Autolysate had strong ACE inhibitory activity. The peptide SASVIPVSAVRA isolated from the yak bone hydrolysate also contained the tripeptide above-mentioned (VIP). The ACE inhibitory activity may also be due to the presence of ACE inhibitor VIP in its structure. However, this peptide still needed further tests on SHRs to determine whether its activity remained *in vivo*. It was also necessary to determine the digestion stability and the activity of the peptide after digestion. Therefore, it was discussed about the blood pressure inhibitory effect of the peptide *in vivo* and the simulated digestion results as follows.

### Gastrointestinal digestion of ser-ala-ser-val-ile-pro-val-ser-ala-val-arg-ala (SASVIPVSAVRA) *in vitro*

More and more evidence showed that some bioactive peptides were unstable after digestion (28). Therefore, it is also very important to evaluate the digestive stability of bioactive peptides.

**FIGURE 3:**
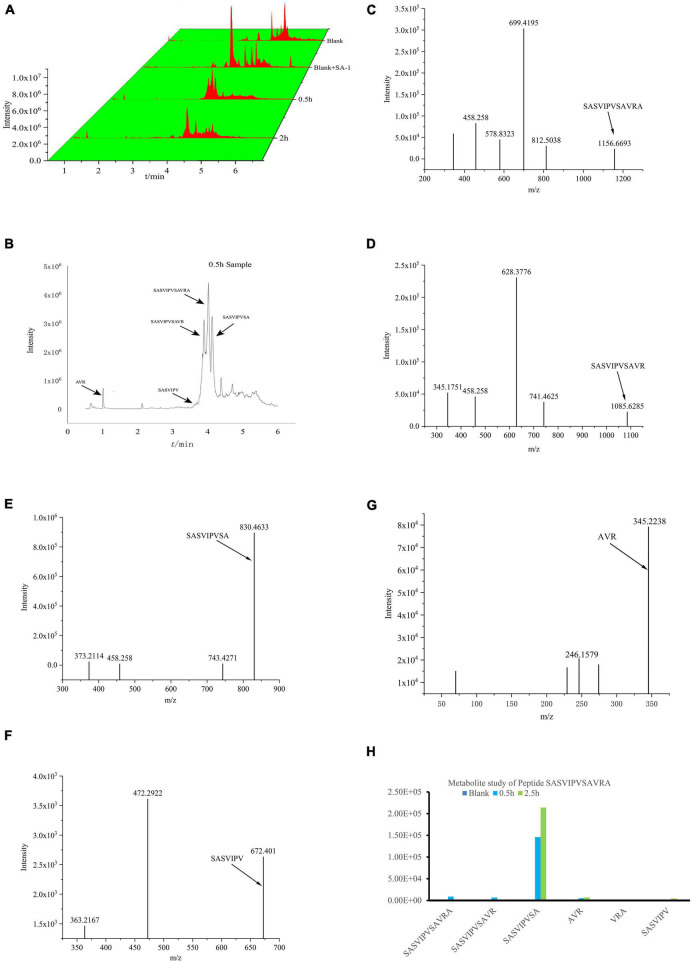
Analysis of peptides SASVIPVSAVRA after digestion. **(A)** 3D waterfall Tic chromatography of the samples. **(B)** Tic chromatography of the 0.5 h samples. **(C)** Ms spectrum of peptide SASVIPVSAVRA. **(D)** Ms spectrum of peptide SASVIPVSAVR. **(E)** Ms spectrum of peptide SASVIPVSA. **(F)** Ms spectrum of peptide SASVIPV. **(G)** Ms spectrum of peptide AVR. **(H)** Peak area of Metabolites in digestion samples.

The IC_50_ of SASVIPVSAVRA before and after digestion were 54.22 and 44.06 μM, respectively. The ACE inhibitory activity after digestion increased about 19%. SASVIPVSAVR, SASVIPVSA, SASVIPV, and AVR were found in the digestion sample (Figure 3). The IC_50_ were 102.3, 144.6, 167.6, 213.2 μM.

There is no significant peak of SASVIPVSAVRA found in the digested samples, indicating SASVIPVSAVRA was easily digested by gastrointestinal tract. SASVIPVSA was the main metabolite. The reason for the increased ACE inhibitory activity after the peptide SASVIPVSAVRA digestion needs further investigation.

As reported by Katayama et al. (29), certain peptides had certain resistance to digestive proteases *in vivo* and could still show strong biological activity. In addition, Long chain peptides are more easily digested into many short peptides, and active peptides are more likely to appear (30). Therefore, in the future, we will further study the transport of peptide in human intestinal CaCo-2 cell monolayers to determine the absorption of the peptide *in vitro*.

### The angiotensin-converting enzyme inhibition patterns

**FIGURE 4:**
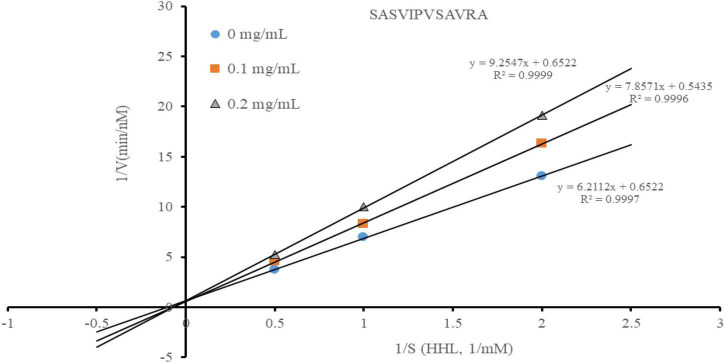
Line weaver-Burke plot of ACE activity in the presence of the peptide.

Most peptides are in a competitive ACE inhibition pattern, while a small part of them are in a non-competitive ACE inhibition pattern (31). According to the Lineweaver-Burk plots, the ACE inhibition pattern of the peptide was estimated (Figure 4). The increased Km and constant Vmax indicated that SASVIPVSAVRA acted as a competitive ACE inhibitor. This suggested that SASVIPVSAVRA might competitively bind to the catalytic site of ACE to prevent ACE from binding to the substrate (32). Also, Cheung et al. (33) found those peptides containing hydrophobic C-terminal amino acids (such as alanine, phenylalanine and tyrosine) generally performed a competitive inhibition pattern. From our results, the peptide SASVIPVSAVRA had alanine residues at the C-terminal, respectively. This may offer an explanation of why SASVIPVSAVRA exhibited a competitive inhibitor.

### Molecular docking simulation

**FIGURE 5:**
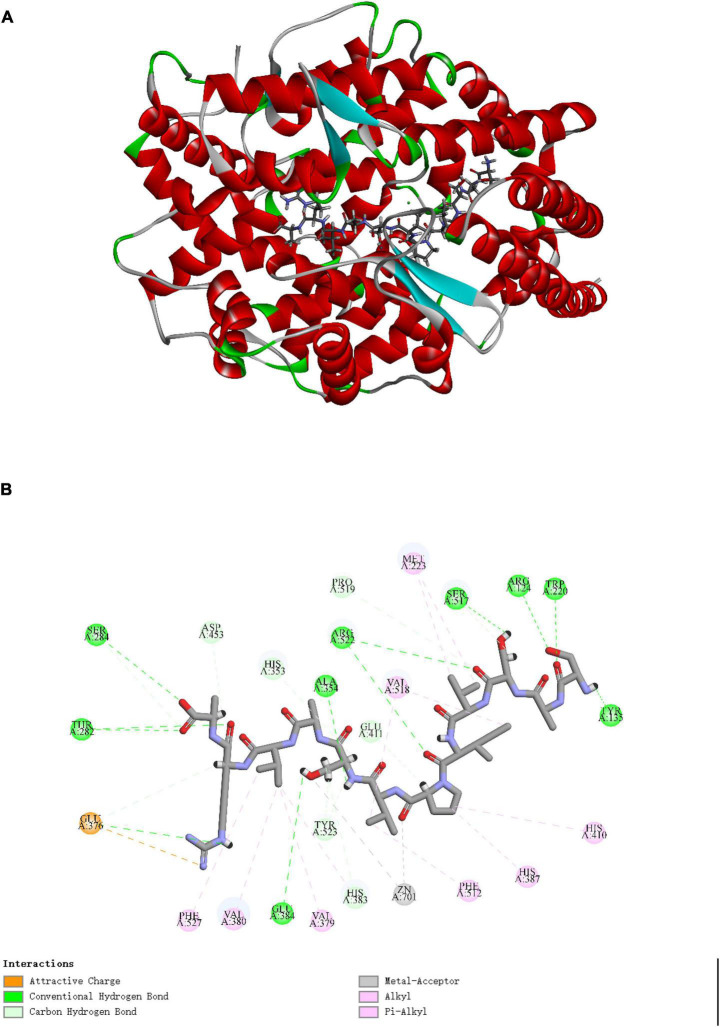
Diagram of SASVIPVSAVRA binding with ACE. **(A)** The optimal docking structure diagram of SASVIPVSAVRA binding with ACE (PDB: 1O8A). **(B)** The 2D diagram of the interaction between SASVIPVSAVRA and ACE amino acid residues.

Molecular docking is to explore the binding interactions effect of peptide on ACE. Figure 5A showed the best posture for docking peptide SASVIPVSAVRA at the active site of ACE through docking simulation. The mutual binding energy was 134.935 kJ/mol. The docking structure Figure 5B showed that Van der Waals forces, hydrogen bonds and metal receptor were the major binding forces in the interaction between SASVIPVSAVRA and ACE.

The peptide existed in the ACE active site’s narrow channel and formed 9 hydrogen bonds with ACE. It indicated that the peptide had strong interaction forces with ACE (34). Specifically, the peptide established hydrogen bonds with ACE residues Arg124, Tyr135, Trp220, Thr282, Ser284, Ala354, Glu384, Ser517, and Arg522, which may be the key residues in ACE binding (35). The active center of ACE comprises zinc ions and three primary active capsules (S1, S2, S3). More studies have shown that high ACE inhibitory activity peptides can directly interact with the ACE Zn(II) (36). This result may offer an clarification of why SASVIPVSAVRA displayed high ACE inhibitory activity.

### Antihypertensive effects of ser-ala-ser-val-ile-pro-val-ser-ala-val-arg-ala (SASVIPVSAVRA) *in vivo*

The antihypertensive effect of peptide SASVIPVSAVRA was evaluated by measuring the changes in SBP in the SHRS within 8 h after administration.

**FIGURE 6:**
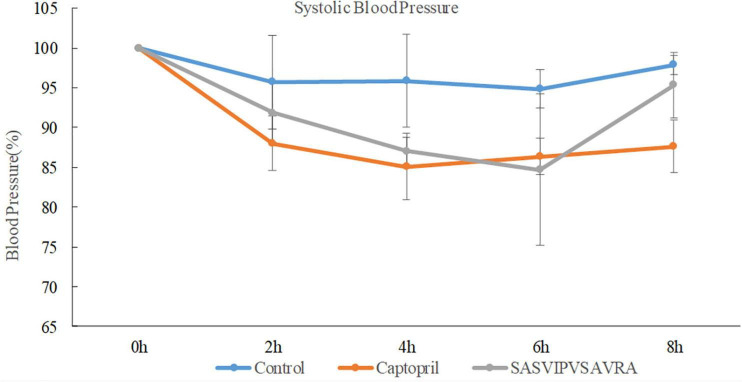
The effects of distilled water, captopril and SASVIPVSAVRA on systolic blood pressure of SHRs.

The systolic blood pressure of the negative control group (sterilized water) had no significant change after administration (Figure 6). The SASVIPVSAVRA group and the captopril group significantly reduced the SBP of SHRs (Figure 6). A significant antihypertensive effect had been observed within 8 h of administration of the SASVIPVSAVRA peptide. Antihypertensive activity peaked at the 6th hour after administration. When the oral dose was 30 mg/kg, the maximum reduction of systolic blood pressure in the sample group and the positive control group was 15.3 and 14.9%, respectively. It was shown that ACE peptide and captopril have similar antihypertensive effects *in vivo*, suggesting that ACE peptide and captopril were not directly proportional to their antihypertensive abilities *in vitro* and *in vivo*, This was consistent with the study by Cao et al. (1). Some chemically synthesized drugs have a substantial effect on lowering the blood pressure *in vitro*, but their effects *in vivo* will be weakened (37, 38). Remarkably, Captopril is a chemically designed and commercially used drug, The peptide SASVIPVSAVRA also had a blood pressure lowering effect *in vivo*, so it could provide a reference for the development of new blood pressure lowering drugs. Furthermore, the ACEI peptide IVGRPRHQG from chicken muscle showed similar antihypertensive effect as SASVIPVSAVRA, but required a higher dose of 60 mg/kg BW (39).

The peptide SASVIPVSAVRA had high ACE inhibitory activity both *in vitro* and *in vivo*. The ACE inhibitory activity increased about 19% after digestion, but none of its metabolites showed stronger activity than it, Some hydrophobic amino acids produced during this digestion are worth considering. The relationship between the metabolites of the peptide in blood and effect of antihypertensive effect *in vivo* should be deeply studied through the plasma metabonomics and bioanalysis.

## Conclusion

A novel angiotensin converting enzyme (ACE) inhibitory peptide SASVIPVSAVRA was purified and identified from yak bone in this study. The results show that the peptide has strong ACE inhibitory activity *in vitro* and hypotensive effect *in vivo*, and has the potential to be developed into antihypertensive drugs.

## Data availability statement

The datasets presented in this study can be found in online repositories. The names of the repository/repositories and accession number(s) can be found in the article/supplementary material.

## Ethics statement

All animal procedures used in this trial were approved by the Institutional Animal Care and Use Committee of Hangzhou Medical College (Approval No. ZJCLA-IACUC-20010014).

## Author contributions

YZ and YD contributed to the conception of the study. XG and FB performed the experiment. DY and HL contributed significantly to analysis and manuscript preparation. ZH, CZ, and CW performed the data analyses and wrote the manuscript. XG, J-ML, and YD helped perform the analysis with constructive discussions. All authors contributed to the article and approved the submitted version.

## Footnotes

1
http://www.rcsb.org
